# A Study of the Chemical Composition, Antioxidant Potential, and Acute Toxicity of Bulgarian *Tanacetum vulgare* L. Essential Oil

**DOI:** 10.3390/molecules28166155

**Published:** 2023-08-21

**Authors:** Diana Karcheva-Bahchevanska, Niko Benbassat, Yoana Georgieva, Borislava Lechkova, Stanislava Ivanova, Kalin Ivanov, Velislava Todorova, Lyudmil Peychev, Zhivko Peychev, Petko Denev

**Affiliations:** 1 Department of Pharmacognosy and Pharmaceutical Chemistry, Faculty of Pharmacy, Medical University-Plovdiv, 4002 Plovdiv, Bulgaria; 2 Department of Pharmacology, Toxicology and Pharmacotherapy, Faculty of Pharmacy, Medical University-Plovdiv, 4002 Plovdiv, Bulgaria; 3 Department of Medical Informatics, Biostatistics and E-Learning, Faculty of Public Health, Medical University-Plovdiv, 4002 Plovdiv, Bulgaria; 4 Laboratory of Biologically Active Substances, Institute of Organic Chemistry with Centre of Phytochemistry—Bulgarian Academy of Sciences, 4000 Plovdiv, Bulgaria

**Keywords:** *Tanacetum vulgare* L., essential oil composition, GC/MS, antioxidant activity, in vivo acute toxicity

## Abstract

Common tansy (*Tanacetum vulgare* L.) is a plant with medicinal properties that has traditionally been used in folk medicine for its anthelmintic, antispasmodic, and choleretic effects, for the treatment of diarrhea and digestive problems, and externally, as an insecticide in veterinary practices. In the current study, we investigated, for the first time, the chemical profile and antioxidant activity of essential oil from a wild population of *T. vulgare* L. growing in Bulgaria. Common tansy essential oil (EO), which is rich in bicyclic monoterpenes, was obtained using hydrodistillation and characterized by using gas chromatography–mass spectrometry (GC-MS). Thirty-seven compounds were identified in Bulgarian tansy EO. Among the major constituents were oxygenated monoterpenes, including compounds such as camphor (25.24%), *trans*-chrysantenyl acetate (18.35%), *cis*-verbenol (10.58%), thujone (6.06%), eucaliptol (5.99%), and α-campholenal (5.98%). The analysis results identified the essential oil from *T. vulgare* L. grown in the western Rhodope Mountains of Bulgaria as the camphor chemotype. Furthermore, its antioxidant activity was analyzed using the oxygen radical absorbance capacity (ORAC) method and was found to be 605.4 ± 49.3 µmol TE/mL. The essential oil was also tested for single-dose acute toxicity on Wistar rats and was found to be non-toxic by oral administration. The mean lethal dose by intraperitoneal administration was LD_50_ i.p. = 14.9 g/kg body weight. The results of the conducted study can serve as a basis for the evaluation and subsequent exploration of other pharmacotherapeutic effects of the essential oil obtained from the inflorescences of the Bulgarian species *T. vulgare* L.

## 1. Introduction

The genus *Tanacetum* contains over 200 species that belong to the Asteraceae (Compositae) family. In Bulgaria, the *Tanacetum* genus is represented by six species [1], and only *Tanacetum parthenium* L. (feverfew) is included in the *European Pharmacopoeia*. *Tanacetum vulgare* L. (syn. *Chrysanthemum vulgare* L.), known as common tansy, is a perennial flowering herbaceous plant common in the northern hemisphere that grows as a wild weed along roadsides, waste places, and hedgerows, and can reach up to 1 m in height [2]. The flower heads are yellow, composed only of tube-shaped flowers, and lacking strap-shaped flowers. The leaves are divided compound.

The genus is an abundant producer of various secondary metabolites such as sesquiterpenoids, flavonoids, and essential oils [3]. The chemical composition of *Tanacetum* species has been a subject of attention since terpenoids, particularly sesquiterpene lactones, were thought to possess different biological activities. *Tanacetum parthenium* L. contains a sesquiterpene lactone parthenolide that belongs to the germacranolide class and has antimigraine potential [4]. The major compounds in the crude extract have been determined to be hydroxycinnamoyl quinic acids and flavonoid derivatives. The main flavonoid aglycone in tansy has been identified as luteolin [5] and the presence of flavonoids, phenolic acids, and terpenoids in *T. vulgare* L. extracts has been confirmed by Ak et al., 2021 [6]. Due to the high content of bioactive compounds, tansy extracts display various biological activities. For example, exposure to tansy extract has been reported to decrease the viability of breast cancer cell lines in a dose- and time-dependent manner [7]. Cytotoxic activity against in vitro cultured cancer and healthy cell lines has been reported for eudesmanolides isolated from flowers of *T. vulgare* L. growing in Sicily [8]. Vasileva et al. tested tansy extracts and fractions on cell lines in vitro [5]. Ethyl acetate extracts showed the highest selective activity towards tumor cells. Tansy essential oil showed strong activity against fungi and Gram-negative *E. coli* and *E. cloacae* [2]. Cote et al. assessed the anti-inflammatory, antibacterial, antioxidant, and cytotoxic activities of *T. vulgare* L. collected from Canada [9]. Furthermore, a dose-dependent in vivo hepatoprotective and choleretic effect of dried extract from *T. vulgare* flowers was established by Mishchenko et al. [10]. Larocque et al. investigated the effects of tansy EO on *Choristoneura rosaceana* and found that the inclusion of the essential oil in the diet of the oblique banded leaf roller affected female pupal weight [11]. However, in vivo studies about *T. vulgare* L. EO are limited. Most of the studies are focused on the extracts rather than on the essential oil. Researchers are continuing to discover new and effective sources of antioxidants for the treatment of a range of diseases. In this line of thought, plants can be considered to be an excellent source of antioxidants because of the many secondary metabolites that scavenge radicals. The intake of natural antioxidants such as essential oils from plants has been associated with reduced risk of various degenerative diseases such as cardiovascular disease, diabetes, dementia, and cancer [12]. There are only a few studies on the antioxidant activity of tansy, mostly conducted on extracts, with the DPPH method used and only two studies on the isolated essential oil, with DPPH, ABTS, FRAP and cell-based assays selected [9,13,14,15,16,17]. 

Plant chemotypes are differentiated according to the amount of the major component in their essential oil. The number of chemotypes further increases based on the amounts of the second and even the third major components of an essential oil. The widespread thujone chemotype of tansy essential oil has been established in 15 countries on the European and American continents. Thujone is considered to be a neurotoxicant, although there have recently been differing opinions on the matter. Thujone is contained in widely varying amounts in the essential oils of a number of medicinal plants including wormwood, tansy, sage, thyme, and rosemary [18]. In addition to α (*cis*)-thujone and β (*trans*)-thujone, the following chemotypes of tansy essential oil have also been reported: camphor, α-pinene, borneol, γ-terpinene, 1,8-cineole, and artemisia ketone, *trans*-chrysantenyl acetate, chrysanthenone, chrysanthenone oxide, bornyl acetate, sabinene, thujyl acetate, thujyl alcohol, umbellulone, isopinocamphone, piperitone, *trans*-dihydrocarvone, lyratol, lyratyl acetate, davanone, and germacrene D [19]. In 2001, the sesquiterpene lactone profile of the Bulgarian *Tanacetum* species was studied by Todorova and Evstatieva [1]. However, there are no data on the phytochemical composition of the essential oil from a wild population of tansy growing in Bulgaria, as well as its antioxidant properties and toxicity. Therefore, the aim of the present study was to investigate the chemical composition of the obtained essential oil, its antioxidant activity and in vivo acute toxicity. In this paper, we describe the camphor chemotype of the essential oil from a wild population of *T. vulgare* growing in the western Rhodope Mountains of Bulgaria. This will assist in exploring the potential of this species as an essential-oil-bearing plant of medicinal and economic importance. 

## 2. Results and Discussion

### 2.1. Volatile Organic Compounds and Antioxidant Capacity of Tanacetum vulgare L. Inflorescences Essential Oil

**Figure 1:**
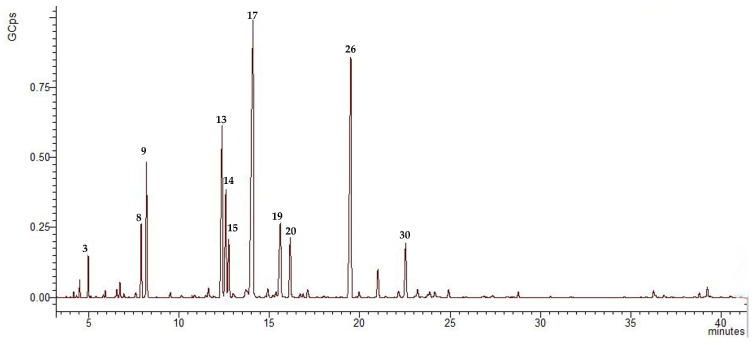
Chromatogram of essential oil obtained from a wild population of *Tanacetum vulgare* L. growing in Bulgaria, compounds derived from GC-MS analysis. The main compounds of the EO derived from the inflorescences are presented as follow: 3. camphene; 8. *p*-cymene; 9. eucalyptol (1,8-cineole); 13. *cis*-verbenol; 14. β-thujone; 15. chrysantenone; 17. camphor; 19. α-campholenal; 20. terpinen-4-ol; 26. *trans*-chrysantenyl acetate; 30. bornyl acetate. GCps—giga-counts per second.

The extracted essential oil of *T. vulgare* L. obtained by hydrodistillation was yellow to pale green in color and had a distinct odor. The extraction yield obtained after 4 h was 1.2% (*w*/*w*). The oil was analyzed by GC-MS and thirty-seven volatile organic compounds were identified, representing 93.23% of the total essential oil components detected. The chromatogram of *T. vulgare* L. essential oil is shown in Figure 1.

The chemical composition of the essential oil was rich in compounds from the following terpene classes: monoterpene hydrocarbons (6.12%), oxygenated monoterpenes (79.55%), and oxygenated sesquiterpenes (1.17%). The main EO components were oxygenated monoterpenes comprising compounds such as camphor (25.24%), *trans*-chrysantenyl acetate (18.35%), and *cis*-verbenol (10.58%), as well as β-thujone (6.06%), eucaliptol (5.99%), and α-campholenal (5.98%). Among the monoterpene hydrocarbons, the aromatic monoterpene *p*-cymene (3.16%) was in the highest concentration, followed by camphene (1.84%) and α-pinene (0.52%). In regard to oxygenated sesquiterpenes, they were the least represented with the main compounds being β-eudesmol (0.47%) and spathulenol (0.36%). Table 1 presents the chemical composition of the essential oil as determined by GC-MS analysis with formulas, RIs, and relative percentage amounts of the compounds.

Essential oil quality and yield are influenced by many factors, such as soil pH, chemotype or subspecies, plant part selection, harvesting season, plant phenology, drying conditions, and extraction methods [20]. Tansy herb (*Tanaceti herba*), collected at the full flowering stage, usually contains from 0.1 to 1.1% of essential oil [19]. In our study, the essential oil content was higher (1.2 ± 0.07%), which can be explained by the fact that the oil was obtained from the inflorescences (*Tanaceti flos*) and not from the whole aerial parts. The main compounds of EO from inflorescences of wild populations of common tansy in Finland are the monoterpenoids camphor (435 mg/g), *trans*-chrysanthenyl acetate (194 mg/g), and eucalyptol (98 mg/g) [21]. In the data we obtained, the first two predominant compounds in the mixture were camphor and *trans*-chrysantenyl acetate, the sum of which accounted for 43.59% of the total essential oil components. The chemical composition data of essential oil obtained from the aerial parts of *T. vulgare* in Northern Quebec, Canada corresponded with ours regarding the leading compound, i.e., camphor, with a value of 30.48% [9]. In contrast, for the tansy species distributed in Sicily, Formisano et al. reported the highest thujone content (34.5%) and the absence of camphor in samples. In addition, the authors observed that the essential oil from the flowers was richer in β-thujone, while the essential oil from the aerial parts contained a greater amount of α-thujone [22]. In our case, the essential oil was derived from inflorescences, and it contained β-thujone (6.06%); α-thujone was not detected. Plants and the subsequent essential oil obtained from *T. vulgare* L. can have different chemotypes. The widespread thujone chemotype of tansy essential oil has been established in 15 countries of the European and American continents. This chemotype is not as common in the EO samples of plants from the Vilnius district, as well as from the Tsigov Chark area that we investigated [19]. According to Devrnja et al., tansy extracts and low thujone essential oil may be promising in antimicrobial applications and food preservation [2]. Based on the chemical composition determined from three independent samples in triplicate, we classify the Bulgarian tansy essential oil as the camphor chemotype. Camphor is a bicyclic monoterpene ketone that was originally distilled from the bark of the camphor tree and is now synthesized from terpentine oil. It has effects that range from mild central nervous system excitation to generalized seizures. Camphor is an ingredient in some topical medicines for the treatment of musculoskeletal pain or flu-like symptoms. It is marketed as an analgesic, an antitussive, and an antipruritic [23]. Based on the identical results for chemical composition determined from the three samples with triplicates for each sample, one of the three samples was randomly selected for further assays of antioxidant activity and acute toxicity.

Natural antioxidants stimulate cellular protection and reduce oxidative damage or stress associated with diseases such as atherosclerosis, cardiovascular disease, neurodegenerative disease, cancer, and the aging process [6,12]. The antioxidant potential of an essential oil depends on its composition. Phenolic compounds and other secondary metabolites with conjugated double bonds usually show significant antioxidant properties [15]. The antioxidant activity of the Bulgarian tansy EO was determined using the oxygen radical absorbance capacity (ORAC) method, which relies on the mechanism of hydrogen atom transfer. The lipophilic modification of the assay measures the antioxidant scavenging function of lipophilic antioxidants against peroxyl radical induced by 2,2′-azobis (2-amidinopropane) dihydrochloride (AAPH) at 37 °C. In our study, the tansy essential oil ORAC value was 605.4 ± 49.3 µmol TE/mL. According to the literature data, essential oil from *T. vulgare* L. has demonstrated antioxidant activity by significantly inhibiting tert-butylhydroperoxide-induced oxidation of 2′,7′-dichlorofluorescin (DCFH oxidation) with an IC_50_ of 51 µg mL^−1^ [9]. Shaparov et al. also reported moderate antioxidant activity of the essential oil from *T. vulgare* L. using in vitro ABTS, DPPH, and FRAP assays [15]. In most studies, the antioxidant activity has been determined on tansy extracts rather than on the resulting essential oil. The antioxidant potential of *Tanacetum* species extracts may be primarily due to the presence of phenolic acids. Caffeic acid derivatives are known for their high antioxidant activity [14]. In some cases where the essential oil has been tested for this activity, a method other than ORAC has been used, and therefore, it is difficult to compare the results we obtained. The antioxidant activity of essential oil is often attributed to terpenoids such as the aromatic monoterpenes carvacrol and thymol, as well as the phenylpropane derivative eugenol [9]. However, to the best of our knowledge, this is the first time that the antioxidant capacity of essential oil derived from the Bulgarian tansy has been reported.

### 2.2. Evaluation of Single Dose Acute Toxicity (LD_50_)

Acute toxicity results are presented in Table 2 for the oral and in Table 3 for the intraperitoneal route of administration. The Litchfield and Wilcoxon method was used to calculate the mean Lethal Dose for i.p. administration of the EO because when administered p.os no mortality was detected [24]. LD_50_ i.p. =14.9 g/kg body weight (b.w). (12.7 ÷ 17.5); χ^2^ = 1.14; *p* < 0.05; S_function_ = 1.28.

When essential oil was administered p.os at the above doses, no symptoms of poisoning were found, and no mortality occurred among the experimental animals up to the 24 h.

The application of tansy extracts is primarily external as antiparasitic agents and should be used with special care when consumed by humans and animals due to the toxic effects of β-thujone. Symptoms of tansy EO poisoning are attributed to the same toxic ketone content and include severe gastritis, intense spasms and convulsions, liver and brain damage, rapid and weak pulse [25,26]. Despite individual reports that EO high in thujone induces the toxic manifestations described above, there is no systematic evaluation of the toxic effects of tansy [27]. It would be interesting to explore the possibilities of different chemotypes in which this bicyclic monoterpene is in a lower concentration, as well as their efficacy and safety. It would be also interesting to explore the chemical composition of cultivated *T. vulgare* L. EO [28]. The current investigation showed that the EO of the Bulgarian population of *T. vulgare* L., used in traditional medicine for a number of medical conditions, is non-toxic when administered to rats as a single oral dose and relatively non-toxic when administered to rats as a single intraperitoneal dose, as the calculated LD_50_ was 14.9 g/kg b.w. for the intraperitoneal dose. The LD_50_ has been found to be between 5 and 15 g/kg, which corresponds to toxicological level 5 on the Hodge and Sterner scale, i.e., it is practically non-toxic to humans [29]. In the last two decades, research on herbs with potential use in medicine is thriving [30,31,32,33]. The antioxidant potential and the non-toxicity of *T. vulgare* L. EO demonstrated in the current study would be an important key point for future research.

## 3. Materials and Methods

### 3.1. Chemicals and Reagents

The following hydrocarbons were used to determine the retention indices (RIs): nonane (99%), decane (≥99%), undecane (≥99%), dodecane (99%), tridecane (≥99%), tetradecane (≥99%), hexadecane (≥99%), heptadecane (99%), octadecane (99%), nonadecane (99%), eicosane (99%), heneicosane (≥99.5%), tricosane (99%), tetracosane (99%), pentacosane (99%), octacosane (99%), and triacontane (99%), and they were purchased from Merck KGaA (Darmstadt, Germany). GC-grade hexane was used for diluting the essential oils, purchased from Thermo Fisher Scientific GmbH (Bremen, Germany). Fluorescein (3′,6′-dihydroxy-3H-spiro [2-benzofuran-1,9′-xanthen]-3-one) (CAS 2321-07-5), AAPH (2,2′-azobis-(2-methylpropionamidine)-dihydrochloride) (CAS 2997-92-4), and trolox (6-hydroxy-2,5,7,8-tetramethylchroman-2-carboxylic acid) (CAS 53188-07-1) were purchased from Sigma-Aldrich (Darmstadt, Germany).

### 3.2. Plant Material

The inflorescences (*Tanaceti flos*) of wild-grown *Tanacetum vulgare* L. were collected in July 2021. The usable plant parts were gathered in the western Rhodope Mountains of Bulgaria, Tsigov Chark, Batak, located at 1100 m altitude (41°52′47.4″ N 24°09′36.7″ E). The plant was authenticated by the authors according to Flora of the Republic of Bulgaria [34]. The collection of the inflorescences was carried out in the phase of full flowering and then were dried at room temperature (20–25 °C).

### 3.3. Isolation of Essential Oil

The essential oil of the air-dried plant material (inflorescences) was determined by performing hydrodistillation for 4 h, using *n*-hexane/water as the solvent and a Clevenger-type apparatus, according to the *European Pharmacopoeia* method. The collected essential oil (1.2 ± 0.07% yield based on the dry weight of the inflorescences, *w*/*w*) was dried over anhydrous sodium sulfate and stored in dark glass vials at 4 °C. Using this approach three independent samples of EO were prepared. After conducting a GC-MS analysis, it was established that all samples were camphor type. The chemical composition of the samples was identical. Therefore, only one of the samples was used for further analysis of antioxidant activity and acute toxicity.

### 3.4. Gas Chromatography-Mass Spectrometry (GC-MS) Analysis

The EO diluted with hexane 1:50 (*v*/*v*) was analyzed using gas chromatography-mass spectrometry. The GC-MS analysis was performed on a Bruker Scion 436-GC SQ MS system (Bremen, Germany), fitted with a fused silica Bruker BR-5ms capillary column (15 m × 0.25 mm, 0.25 μm film thickness). The ionization voltage for the mass was 70 eV, and the spectral range was 50–250 *m*/*z* in full scan mode. The oven temperature was held at 45 °C for 1 min, increased to 90 °C at a rate of 2 °C/min, and then, programmed to 140 °C at a rate of 3 °C/min. Finally, the temperature was increased to 250 °C at a rate of 30 °C/min and held for 1 min. The carrier gas was helium, with a flow rate of 1.0 mL/min; the temperatures of the detector and injector were adjusted to 300 and 250 °C, respectively. The injector was split/splitless, the injection volume was 1 μL, and the split ratio was 1:20. The compounds were identified by comparing their mass spectra and RIs following the Kovats method (determined by a series of *n*-alkanes (C_9_–C_30_) with linear interpolation). The resulting RIs were compared with those from the Wiley NIST11 Mass Spectral Library and literature data [35,36].

### 3.5. Lipophilic Oxygen Radical Absorbance Capacity (ORAC) Assay

The lipophilic oxygen radical absorbance capacity (ORAC) assay was performed according to the procedure described by Huang et al. (2002) [37]. Briefly, working solutions of fluorescein (63 nM) were prepared by dissolving fluorescein disodium salt in phosphate buffer (75 mM, pH = 7.4). The total reaction mixture volume was 200 μL and all solutions were prepared in a phosphate buffer (75 mM, pH = 7.4). Seven percent solution (*w*/*v*) of randomly methylated β-cyclodextrins (RMCDs) in a 50% acetone-H_2_O mixture was used as a solubility enhancer of lipophilic samples. Then, 170 μL of fluorescein solution (53.6 nM final concentration) and 10 μL of the sample were placed in the wells of a 96 microplate and incubated at 37 °C directly in the FLUOstar plate reader for 20 min. After the incubation, 20 μL of AAPH (51.5 mM final concentration) was added rapidly using a multichannel pipette to start the reaction. The fluorescence was recorded every minute and the microplate was automatically shaken prior to each reading. A blank using 7% RMCD solution, instead of the antioxidant and calibration solutions of Trolox in 7% RMCDs solution (6.25, 12.5, 25, and 50 μM) as an antioxidant, was also carried out in each assay. The protective effect of an antioxidant was measured by assessing the area under the fluorescence decay curve (AUC) and comparing it to that of a blank in which no antioxidant was added. The final ORAC values were calculated by using a regression equation between the Trolox concentration and the net area under the curve (AUC). The net AUC corresponding to the sample was calculated by subtracting the AUC corresponding to the blank. The measurement was performed in six repetitions on a FLUOstar OPTIMA fluorimeter (BMG LABTECH, Offenburg, Germany), an excitation wavelength of 485 nm and emission wavelength of 520 nm were used. The ORAC values were calculated as means ± standard deviation (SD; n = 6) and expressed as μM Trolox equivalents per milliliter (µmol TE/mL) oil.

### 3.6. Single Dose Acute Toxicity Test

Sixty male Wistar rats (180.0 ÷ 200.0 g b.w.), equally divided into 10 groups, were administered a single dose of tansy EO by two routes of administration—oral (p.os) and intraperitoneal (i.p.). Groups № I, II, III, IV and V were administered EO p.os by gastric tube at doses of 5 g/kg b.w., 10 g/kg b.w., 20 g/kg b.w., 30 g/kg b.w. and 50 g/kg b.w., respectively. Groups № VI, VII, VIII, IX and X were treated i.p. with doses of 1.0 g/kg b.w., 1.5 g/kg b.w., 1.6 g/kg b.w., 1.8 g/kg b.w. and 2.0 g/kg b.w., respectively. General behavior and mortality were monitored up to the 24 h.

The animals were housed in the university vivarium and kept under standard laboratory conditions: 12:12 h (light/dark) cycle, temperature 23 ± 1 °C, receiving water and laboratory chow *ad libitum*. Permission to use animals in the experiment was obtained from the Food Safety Agency at the Bulgarian Ministry of Agriculture and Food (No. 238/2019, valid until 11 September 2024) and the study was formally approved by the Ethical Committee on Human and Animal Experimentation of the Medical University-Plovdiv. All procedures were conducted in accordance with the European Community Council directives 86/609/EEC. The results for mean Lethal Dose LD_50_-values were evaluated according to Litchfield and Wilcoxon, by *p* < 0.05 [24].

## 4. Conclusions

The chemical profile of the essential oil from a wild population of *T. vulgare* L. growing in Bulgaria was investigated, for the first time, using GC-MS, and its antioxidant capacity was assayed by the ORAC method. The EO was determined to be the camphor chemotype, with the main components being oxygenated monoterpenes and the main compounds being camphor (25.24%), *trans*-chrysantenyl acetate (18.35%), and *cis*-verbenol (10.58%), comprising more than half of the volatile mixture studied. The antioxidant capacity (605.4 ± 49.3 µmol TE/mL) indicated the high scavenging ability of the essential oil towards peroxyl radicals. Our results demonstrated no toxicity associated with the use of tansy EO on Wistar rats. The mean Lethal Dose was LD_50_ i.p. = 14.9 g/kg b.w. (12.7 ÷ 17.5). The documented data contribute to the safety and better understanding of using *T. vulgare* L. as a plant-derived nutrition supplement. The results of this study could serve as a basis for the evaluation of other pharmacotherapeutic activities of the essential oil obtained from the Bulgarian species *T. vulgare* L.

## Figures and Tables

**Table 1:** Volatile organic compounds in essential oil obtained from wild populations of *Tanacetum vulgare* L. growing in Bulgaria, where tr = trace (<0.05%), ND = not detected.

No. ^a^	Compound ^b^	RI ^c^	Molecular Formula	Class of Compound	% of Total ^d^
1	α-Thujene	932	C_10_H_16_	MH	tr
2	α-Pinene	937	C_10_H_16_	MH	0.52
3	Camphene	947	C_10_H_16_	MH	1.84
4	β-Pinene	966	C_10_H_16_	MH	0.25
5	Yomogi alcohol (3,3,6-Trimethyl-1,4-heptadien-6-ol)	990	C_10_H_18_O	O	0.14
6	Mesitylene	999	C_9_H_12_	O	0.27
7	β-Phellandrene	1002	C_10_H_16_	MH	0.11
8	*p*-Cymene	1008	C_10_H_14_	MH	3.16
9	Eucalyptol (1,8-cineole)	1020	C_10_H_18_O	MO	5.99
10	α-Terpinene	1030	C_10_H_16_	MH	0.24
11	γ-Terpinene	1049	C_10_H_18_O	MO	0.11
12	Terpinolene	1076	C_10_H_16_	MH	tr
13	*cis*-Verbenol	1117	C_10_H_16_O	MO	10.85
14	β-Thujone	1118	C_10_H_16_O	MO	6.06
15	Chrysantenone	1120	C_10_H_14_O	MO	2.94
16	*p*-Menth-2-en-1-ol	1122	C_10_H_18_O	MO	0.28
17	Camphor	1128	C_10_H_16_O	MO	25.24
18	Pinocarvone	1159	C_10_H_14_O	MO	0.49
19	α-Campholenal	1135	C_10_H_16_O	O	5.98
20	Terpinen-4-ol	1156	C_10_H_18_O	MO	3.50
21	*p*-Cymene-8-ol	1180	C_10_H_14_O	MO	0.19
22	Myrtenal	1185	C_10_H_14_O	MO	0.21
23	α-Terpineol	1188	C_10_H_18_O	MO	0.51
24	Myrtenol	1190	C_10_H_16_O	MO	0.14
25	Verbenone	1203	C_10_H_14_O	MO	tr
26	*trans*-Chrysantenyl acetate	1236	C_12_H_18_O_2_	MO	18.35
27	Carvone	1245	C_10_H_14_O	MO	0.32
28	Isopiperitenone (*p*-mentha-1,8-dien-3-one)	1274	C_10_H_16_O	MO	tr
29	Verbenyl acetate (bicyclo [3.1.1] hept-2-en-4ol)	1282	C_12_H_18_O_2_	MO	0.47
30	Bornyl acetate	1296	C_12_H_20_O_2_	MO	3.38
31	*p*-Cymen-7-ol	1300	C_10_H_14_O	MO	tr
32	Carvacrol	1306	C_10_H_14_O	MO	0.52
33	Aromandrene oxide	1435	C_15_H_24_O	SO	tr
34	Spathulenol	1569	C_15_H_24_O	SO	0.36
35	Caryophyllene oxide	1571	C_15_H_24_O	SO	0.11
36	Longiverbenone	1627	C_15_H_22_O	SO	0.23
37	β-Eudesmol	1640	C_15_H_26_O	SO	0.47
	Terpene classes				
	Monoterpene hydrocarbons (MH)				6.12
	Oxygenated monoterpenes (MO)				79.55
	Sesquiterpene hydrocarbons (SH)				ND
	Oxygenated sesquiterpenes (SO)				1.17
	Others (O)				6.39
	Total identified				93.23

^a^ In order of elution on BR-5ms column. ^b^ Compounds identified based on RI and MS. ^c^ Calculated retention indices using the Kovats formula. ^d^ The results show the mean of triplicates for each of the three samples, the standard error of the mean not exceeding 2% of it and removed to simplify reporting.

**Table 2:** Results for LD_50_ determination of *T. vulgare* L. essential oil in rats, oral administration.

Group	Dose (g/kg b.w.)	D/T	Dead Rats (%)	Symptoms
I	5	0/6	0	None
II	10	0/6	0	None
III	20	0/6	0	None
IV	30	0/6	0	Hypoactivity. Short twists of the body.
V	50	0/6	0	As above plus rapid breathing. No mortality and no symptoms of intoxication.

None—no symptoms observed up to 24 h. D/T—number of dead rats/number of treated rats.

**Table 3:** Results of LD_50_ determination of *T. vulgare* L. essential oil in rats, intraperitoneal administration.

Group	Dose (g/kg b.w.)	D/T	Dead Rats (%)	Toxic Effect up to 24 h
VI	1.0	0/6	0	None
VII	1.5	4/6	66.7	Rapid breathing. Exitus of 4 animals, at the 12 h.
VIII	1.6	3/6	50.0	Asthenia. Rapid breathing. Trembling. Exitus of 3 rats at the 10 h.
IX	1.8	5/6	83.3	Behavior as dose of 1.6 g/kg b.w. Breathing difficulty. Exitus of 5 rats.
X	2.0	5/6	83.3	Rapid breathing. Difficulty breathing. Some animals emit a short scream. Exitus of 5 rats.

None—no symptoms observed up to 24 h. D/T—number of dead rats/number of treated rats.

## Data Availability

Not applicable.

## References

[1] Todorova M.N., Evstatieva L.N. (2001). Comparative Study of Tanacetum Species Growing in Bulgaria. Z. Naturforschung C.

[2] Devrnja N., Anđelković B., Aranđelović S., Radulović S., Soković M., Krstić-Milošević D., Ristić M., Ćalić D. (2017). Comparative Studies on the Antimicrobial and Cytotoxic Activities of *Tanacetum vulgare* L. Essential Oil and Methanol Extracts. S. Afr. J. Bot..

[3] Azhar M.A.M., Salleh W.M.N.H.W. (2020). Chemical Composition and Biological Activities of Essential Oils of the Genus Litsea (Lauraceae)—A Review. Agric. Conspec. Sci..

[4] Tassorelli C., Greco R., Morazzoni P., Riva A., Sandrini G., Nappi G. (2005). Parthenolide Is the Component of *Tanacetum parthenium* That Inhibits Nitroglycerin-Induced Fos Activation: Studies in an Animal Model of Migraine. Cephalalgia.

[5] Vasileva A.M., Iliev I.A., Lozanov V.S., Dimitrova M.B., Mitev V.I., Ivanov I.P. (2019). In Vitro Study on the Antitumor Activity of *Tanacetum vulgare* L. Extracts. Bulg. Chem. Commun..

[6] Ak G., Gevrenova R., Sinan K.I., Zengin G., Zheleva D., Mahomoodally M.F., Senkardes I., Brunetti L., Leone S., Di Simone S.C. (2021). *Tanacetum vulgare* L. (Tansy) as an Effective Bioresource with Promising Pharmacological Effects from Natural Arsenal. Food Chem. Toxicol..

[7] Gospodinova Z., Antov G., Angelova S., Krasteva M. (2014). In vitro antitumor potential of Bulgarian *Tanacetum vulgare* L. on human breast adenocarcinoma cells. Int. J. Pharm. Sci..

[8] Rosselli S., Bruno M., Raimondo F.M., Spadaro V., Varol M., Koparal A.T., Maggio A. (2012). Cytotoxic Effect of Eudesmanolides Isolated from Flowers of *Tanacetum vulgare* ssp. Siculum. Molecules.

[9] Coté H., Boucher M.-A., Pichette A., Legault J. (2017). Anti-Inflammatory, Antioxidant, Antibiotic, and Cytotoxic Activities of *Tanacetum vulgare* L. Essential Oil and Its Constituents. Medicines.

[10] Mishchenko O., Kalko K., Zolotaikina M., Gontova T., Mashtaler V., Yurchenko C., Derymedvid L., Pozdniakova A. (2019). Hepatoprotective and choleretic activity of dried extract of *Tanacetum vulgare* flowers. Thai J. Pharm. Sci..

[11] Larocque N., Vincent C., Bélanger A., Bourassa J.-P. (1999). Effects of Tansy Essential Oil from *Tanacetum Vulgare* on Biology of Oblique-Banded Leafroller, Choristoneura Rosaceana. J. Chem. Ecol..

[12] Liguori I., Russo G., Curcio F., Bulli G., Aran L., Della-Morte D., Gargiulo G., Testa G., Cacciatore F., Bonaduce D. (2018). Oxidative Stress, Aging, and Diseases. Clin. Interv. Aging.

[13] Nurzyńska-Wierdak R., Sałata A., Kniaziewicz M. (2022). Tansy (*Tanacetum vulgare* L.)—A Wild-Growing Aromatic Medicinal Plant with a Variable Essential Oil Composition. Agronomy.

[14] Bączek K.B., Kosakowska O., Przybył J.L., Pióro-Jabrucka E., Costa R., Mondello L., Gniewosz M., Synowiec A., Węglarz Z. (2017). Antibacterial and Antioxidant Activity of Essential Oils and Extracts from Costmary (*Tanacetum balsamita* L.) and Tansy (*Tanacetum vulgare* L.). Ind. Crops Prod..

[15] Sharopov F., Braun M., Gulmurodov I., Khalifaev D., Isupov S., Wink M. (2015). Antimicrobial, Antioxidant, and Anti-Inflammatory Activities of Essential Oils of Selected Aromatic Plants from Tajikistan. Foods.

[16] Ivănescu B., Tuchiluș C., Corciovă A., Lungu C., Mihai C.T., Gheldiu A., Vlase L. (2018). Antioxidant, antimicrobial and cytotoxic activity of *Tanacetum vulgare*, *Tanacetum corymbosum* and *Tanacetum macrophyllum* extracts. Farmacia.

[17] Aćimović M., Puvača N. (2020). *Tanacetum vulgare* L.—A Systematic Review. Technol. Eng. Manag. J. Agron. Technol. Eng. Manag..

[18] Committee on Herbal Medicinal Products (2011). Public Statement on the Use of Herbal Medicinal Products Containing Thujone.

[19] Mockute D., Judzentiene A. (2004). Composition of the Essential Oils of *Tanacetum vulgare* L. Growing Wild in Vilnius District (Lithuania). J. Essent. Oil Res..

[20] Mikulášová M., Vaverková Š. (2021). Antimicrobial Effects of Essential Oils from *Tanacetum vulgare* L. and *Salvia officinalis* L., Growing in Slovakia. Nova Biotechnol. Chim..

[21] Korpinen R.I., Välimaa A.-L., Liimatainen J., Kunnas S. (2021). Essential Oils and Supercritical CO_2_ Extracts of Arctic Angelica (*Angelica archangelica* L.), Marsh Labrador Tea (*Rhododendron tomentosum*) and Common Tansy (*Tanacetum vulgare*)—Chemical Compositions and Antimicrobial Activities. Molecules.

[22] Formisano C., Senatore F., Bruno M., Rosselli S., Bellone G., Spadaro V. (2009). Essential Oil Composition of *Tanacetum vulgare* subsp. *siculum* (Guss.) Raimondo et Spadaro (Asteraceae) from Sicily. Nat. Prod. Commun..

[23] Hausner E.A., Poppenga R.H. (2013). Hazards Associated with the Use of Herbal and Other Natural Products. Small Animal Toxicology.

[24] Litchfield J.T., Wilcoxon F. (1949). A simplified method of evaluating dose-effect experiments. J. Pharmacol. Exp. Ther..

[25] Barnes J., Anderson L., Phillipson D. (2007). Herbal Medicines.

[26] Sowa P., Marcinčáková D., Miłek M., Sidor E., Legáth J., Dżugan M. (2020). Analysis of Cytotoxicity of Selected Asteraceae Plant Extracts in Real Time, Their Antioxidant Properties and Polyphenolic Profile. Molecules.

[27] Lahlou S., Israili Z.H., Lyoussi B. (2008). Acute and Chronic Toxicity of a Lyophilised Aqueous Extract of *Tanacetum vulgare* Leaves in Rodents. J. Ethnopharmacol..

[28] Von Cossel M. (2022). How to Reintroduce Arable Crops after Growing Perennial Wild Plant Species Such as Common Tansy (*Tanacetum vulgare* L.) for Biogas Production. Energies.

[29] Grossel S.G., Crowl D.A. (1994). Handbook of Highly Toxic Materials.

[30] Gheraissa N., Chemsa A.E., Cherrada N., Erol E., Elsharkawy E.R., Ghemam-Amara D., Zeghoud S., Rebiai A., Messaoudi M., Sawicka B. (2023). Biochemical Profile and In Vitro Therapeutic Properties of Two Euhalophytes, Halocnemum strobilaceum Pall. and *Suaeda fruticosa* (L.) Forske., Grown in the Sabkha Ecosystem in the Algerian Sahara. Molecules.

[31] Benchikha N., Chelalba I., Debbeche H., Messaoudi M., Begaa S., Larkem I., Amara D.G., Rebiai A., Simal-Gandara J., Sawicka B. (2022). Lobularia Libyca: Phytochemical Profiling, Antioxidant and Antimicrobial Activity Using In Vitro and In Silico Studies. Molecules.

[32] Elshafie H.S., De Martino L., Formisano C., Caputo L., De Feo V., Camele I. (2023). Chemical Identification of Secondary Metabolites from Rhizospheric Actinomycetes Using LC-MS Analysis: In Silico Antifungal Evaluation and GrowthPromoting Effects. Plants.

[33] Amato G., Caputo L., Francolino R., Martino M., De Feo V., De Martino L. (2023). Origanum heracleoticum Essential Oils: Chemical Composition, Phytotoxic and Alpha-Amylase Inhibitory Activities. Plants.

[34] Kozhuharov S., Anchev M. (2012). Flora of the Republic of Bulgaria.

[35] Adams R.P. (2017). Identification of Essential Oil Components by Gas Chromatography/Mass Spectrometry.

[36] NIST Chemistry WebBook, SRD 69. https://webbook.nist.gov/chemistry.

[37] Huang D., Ou B., Hampsch-Woodill M., Flanagan J.A., Deemer E.K. (2002). Development and Validation of Oxygen Radical Absorbance Capacity Assay for Lipophilic Antioxidants Using Randomly Methylated β-Cyclodextrin as the Solubility Enhancer. J. Agric. Food Chem..

